# The efficacy of a resilience-enhancement program for mothers in Japan based on emotion regulation: study protocol for a randomized controlled trial

**DOI:** 10.1186/s40359-019-0344-6

**Published:** 2019-11-06

**Authors:** Hiromi Tobe, Mariko Sakka, Kiyoko Kamibeppu

**Affiliations:** Department of Family Nursing & Global Nursing Research Center, Graduate School of Medicine, The University of Tokyo, 7-3-1 Hongo, Bunkyo-ku, Tokyo, 113-0033 Japan

**Keywords:** Resilience, Child protection, Emotion regulation, Randomized controlled trial, Maternal stress, Maternal anger, Anger management, Child maltreatment, Child emotional abuse, Cognitive behavioral therapy

## Abstract

**Background:**

The demands of daily life often cause mothers high levels of distress and other negative emotions. Anger, including harsh verbal discipline, has been linked to child maltreatment, with long-term adverse effects on a child’s well-being. It is critically important to teach mothers stress management and emotion regulation in addition to parenting skills, but this is yet to be conducted in a formalized manner. Strengthening the multiple protective factors that constitute resilience helps reduce distress. The aim of this study is to evaluate the efficacy of a resilience-enhancement program for mothers.

**Methods:**

We designed a two-arm, parallel, randomized trial with an active control. Mothers and their partners with children between three and six years old will be recruited. Following an online baseline survey, 140 mothers will be randomly allocated to either an intervention or control group. Self-report assessment will be conducted online post-intervention and at a two-month follow-up. The control group will participate in a serious of group discussions. The intervention group will participate in four bi-weekly 120-min sessions of a Cognitive Behavior Therapy-based program designed to enhance resilience, focusing on emotion regulation through cognitive reappraisal. Participants will be encouraged to apply and share the skills they acquire with their partner and children at home. Partners will also be assessed to explore their indirect influence from the mothers. Intention-to-treat analysis will be conducted and the two groups will be compared, applying covariate analysis. The primary outcome of the intervention is improved resilience. Secondary outcomes include improved anger control, self-esteem, cognition of children’s misbehavior, and reduced parental stress.

**Discussion:**

To the best of our knowledge, this study will evaluate the first resilience-enhancement program focused on emotion regulation for mothers in Japan. It will contribute to the existing body of knowledge on building emotional resilience. If the program is found to be effective, it will provide an alternative means to enhance mothers’ resilience against stress and improve their ability to regulate emotion. In so doing, it will offer a way to prevent child maltreatment and protect the mental health of children and families.

**Trial registration:**

UMIN000027232, May 3, 2017.

## Background

Parents experiencing high levels of stress have been found to show a greater frequency of controlling and abusive parenting behaviors [1–3]. Japanese people generally tend to suppress negative emotions, especially anger, towards others [4]; however, when it comes to the expression of anger towards their children, the situation can be quite different. The most frequently reported parental stress response emotion among Japanese mothers with children older than 18 months is anger and irritation, more often than anxiety and depression [5, 6]. Among Japanese mothers of three-year-olds, 62.2% had angry outbursts toward their children in times of stress; of these, 86.7% had resorted to harsh verbal discipline [7].

Harsh verbal discipline has long-term, adverse effects on children’s well-being [8]. Exposure to parents’ verbal aggression during childhood increases the risk for the development of psychopathological issues and is associated with alterations in brain structure [9, 10]. Parents’ harsh verbal discipline has been associated with adolescent conduct problems and depressive symptoms; maternal and paternal warmth did not moderate their longitudinal associations [11]. There is, therefore, an urgent need to support parents in decreasing parental stress and, consequently, child maltreatment.

Japan’s Ministry of Health, Labor and Welfare has recently recognized that the widespread use of verbal aggression and physical punishment against children is a serious concern and launched a public campaign that encourages parents to regulate their emotions and in understanding their children’s developmental stages. However, there is little practical support available to parents to help them understand and implement these suggestions. Appropriate parenting skills training is not enough to prevent maltreatment, and an intervention that decreases stress and increase emotion regulation in combination with parenting skills training might be more effective [6, 12]. As parenting involves multi-faceted stressors, one of the key components of this approach is the enhancement of resilience against stress [13].

In the field of psychology, resilience refers to the “process of effectively negotiating, adapting to, or managing significant courses of stress or trauma” [14]. For many individuals of all ages, resilience is a normative recovery process that occurs in response to stressors. Early resilience research was mostly focused on children [15, 16] and emerged from the investigation of good adaptation and healthy development among children who had experienced adverse life events [17]. Resilience research has been expanding to include larger population groups with specific needs and difficulties, and parental resilience has been identified as a research priority that has been largely neglected [18–20]. Along with resilience against serious traumatic events, the everyday stress of parenting is another area of importance [21, 22].

Studies have shown that resilience positively correlates with lower psychological distress and greater well-being [20], and one of the benefits of applying the resilience framework to a parenting intervention is its emphasis on primary prevention, rather than targeting serious, pre-existing maladjustment [23, 24]. Parental self-efficacy fosters a sense of empowerment and promotes an adaptation process that leads to good-quality parenting [25]. Evidence also suggests that proactive parenting can enhance children’s resilience, increasing their self-confidence [26].

Another benefit of the resilience-enhancement approach is that people are more receptive and willing to participate in programs that support them in developing new skills and emphasize their strengths [27]. With this approach, mother’ strengths can be harnessed to improve their children’s future welfare and to learn from and let go of regrets over their past inappropriate parenting; this has been shown to be effective in driving positive changes [20]. Through the application of this approach, individuals can learn about and strengthen their resilience [16], which ultimately empowers them to change. Since resilience is a multifaceted concept, enhancement requires strengthening multiple protective factors relevant to the target population [20, 28] such as emotion regulation, self-esteem, coping skills, effective problem solving, and family and peer relationships [29, 30].

Emotion regulation (ER) has been suggested as an important factor of resilience [31]. ER refers to “extrinsic and intrinsic processes responsible for monitoring, evaluating, and modifying emotional reactions, their intensive and temporal features in particular, to accomplish one’s goals” [32, 33]. While stressful events often lead to emotional reactions, it is not the event itself that causes a particular emotion, but rather the person’s cognitive appraisal of the event. Cognitive reappraisal (CR) involves reframing an emotionally negative situation in a more positive way to decrease the experience of negative emotion [31, 34]. CR and resilience are positively related, while emotional suppression relates negatively to well-being [35–38].

Multiple stressors experienced by mothers including children’s misbehavior, lack of their partners’ support, and low self-esteem or self-image may impair their resilience. Mothers who hold their children to irrational demands might be prone to anger and the application of harsh disciplinary practices. In order to enhance resilience, parents also need to maintain a positive self-image, accepting their own weaknesses and imperfections [39, 40]. The implementation of CR principles by mothers should improve their resilience and may also decrease their anger and anger expression.

In a randomized controlled trial that evaluated the effectiveness of the resilience-enhancement intervention among university students, the intervention group received four weekly, two-hour sessions to learn the skills necessary to change cognitions related to stressful events. The result showed higher resilience, more effective coping strategies, and higher scores on most of the protective factors targeted [30].

In the first systematic review and meta-analysis of resilience-enhancement programs in adults, the body of randomized trial evidence showed a modest but consistent benefit of these programs in improving a number of outcomes, including resilience, quality of life, self-efficacy, depression, and stress [41].

To the best of our knowledge, no studies have used a randomized controlled trial to evaluate a resilience-enhancement program targeting mothers with a focus on emotion regulation.

### Aim

The aim of this study is to assess the efficacy of a resilience-enhancement program for mothers focused on emotion regulation using a randomized controlled trial.

## Methods/design

**Fig. 1:**
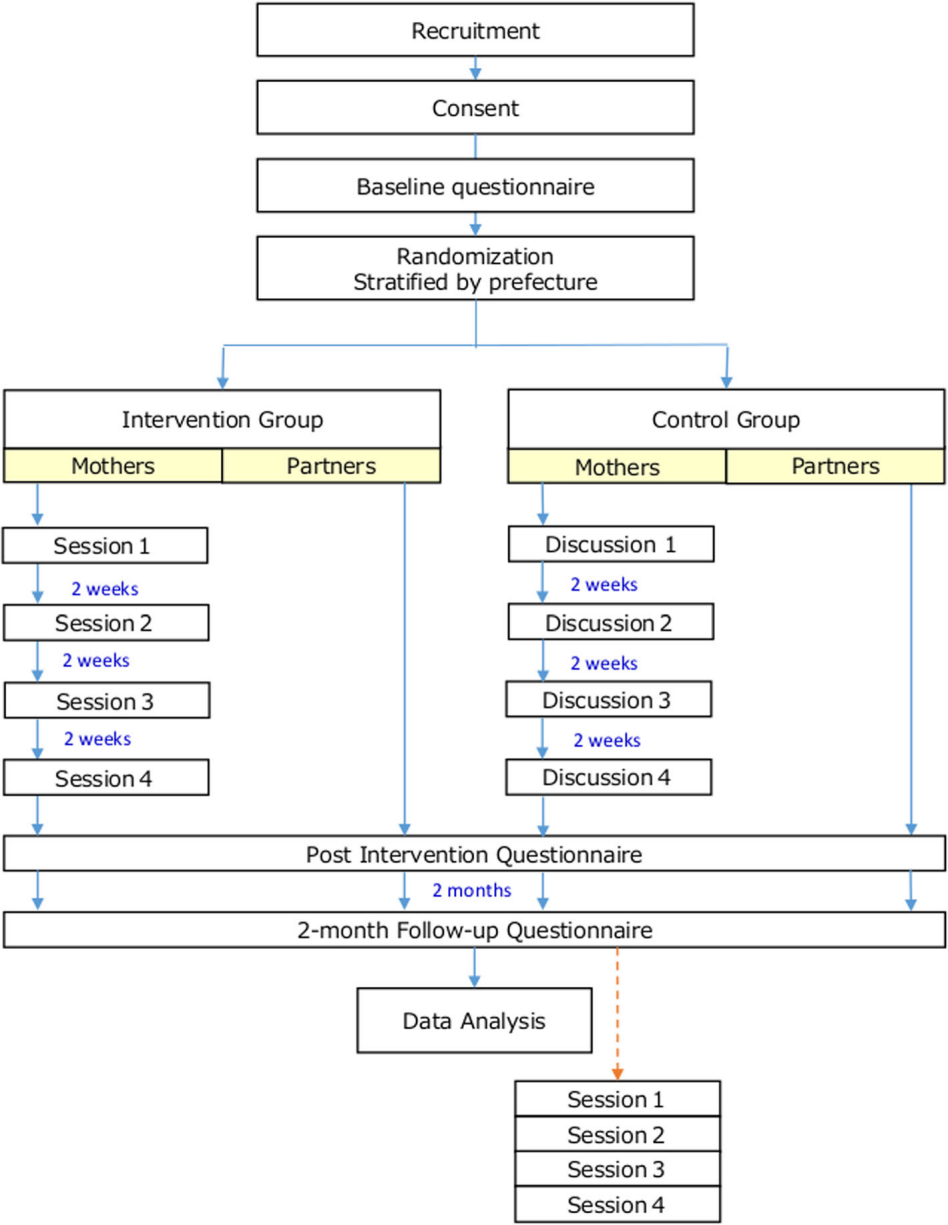
Overview of protocol

This is a non-blinded study with two parallel arms and an active control group (see Fig. 1). The allocation ratio of both groups is 1 to 1.

### Participants

Participants will be recruited at kindergartens, nursery schools, and other facilities where children and their parents gather, in two prefectures in Japan. Recruitment material in the form of flyers and posters will be held to the following criteria:

#### Inclusion criteria

Mothers and their partners who have at least one child between three and six years.

#### Exclusion criteria

1. Those who are diagnosed with a mental disorder
2. Those who do not speak or read Japanese

### Sample size calculation

Sample size was calculated using a statistical power analysis. To detect an effect size of d = 0.62 between the two conditions, with α = .05 (two-tailed) and β = 0.8, 64 dyads in each group are needed. Considering attrition possibility and feasibility, 140 mothers and their partners will be recruited.

### Randomization

After the baseline assessment, dyads will be randomly allocated to either the intervention or comparison group. Stratified-block randomization will be conducted according to the prefecture in which they live.

### Intervention

#### Intervention group

The intervention comprises a group-based training program on Cognitive Behavior Therapy (CBT)-based resilience-enhancement skills. It is structured across four sessions, with each session lasting 120 min. These sessions will be provided biweekly, considering the busy schedules of mothers. The first author, a qualified and experienced teacher of stress and emotion management as well as parenting, will facilitate the sessions. Each group will consist of 6–14 mothers, and they will receive homework assignments to apply their new knowledge to their daily lives and to share the content of the session with their partners and children at home. At the start of each session, participants will share their experiences with each other, including successes, challenges, and changes in themselves and their family members during the two weeks prior. Partners will not participate in the program.

#### Session content

In Session 1, the theme is, “Any emotion is a dear friend.” Participants will learn about resilience and its protective factors. The relationship between parental resilience and its impact on children will be explained. ER will be introduced as one of the protective factors. Participants will learn the meaning and importance of negative emotions, including anger, and why and how they occur. Eight ER skills will be taught in a realistic, applicable way. Using CR skills, participants will learn to recognize the underlying irrational cognitions and unrealistic expectations that may cause negative emotions. The homework assignment is to keep an anger log to monitor anger, to use an anger scale to express anger appropriately to their family, and to encourage their children to do the same.

In Session 2, the theme is, “Worry less and trust more.” Participants will learn how to apply the emotion regulation skills they learned in Session 1 to improve their relationship with their children. The development of children’s brains and emotions will be explained, including the important role of tantrums in the developmental process. In this session, participants will also learn how harsh verbal discipline negatively affects children’s brains and how to avoid it. They will learn how to reframe children’s weaknesses as strengths. The homework assignment is to conduct active listening and to find and verbally appreciate ordinary but appropriate behavior of their children instead of paying attention to and criticizing their misbehaviors and record them. Another assignment is to show empathy and validate children’s feelings when they throw a tantrum without changing the standard or rule.

In Session 3, the theme is, “Difference is strength.” Participants will learn how to improve their relationship with their partners applying the ER skills they learned in the first two sessions. They will discuss how men and women differ in many aspects. They will learn to use CR to recognize their partners’ weaknesses, or the differences between them, as strengths and be introduced to problem-solving strategies. They will be asked to write down five good characteristics of their partners and five things they are grateful for in relation to their partners. The homework assignment will be to tell their partners what they wrote down and to ask them to do some simple tasks, specifically expressing gratitude and appreciation.

In Session 4, the theme is, “I love and trust myself no matter what.” Participants will learn that self-esteem and anger are related, and that self-compassion and self-acceptance are the best skills they could develop – not only for themselves but also for their children’s happiness and resilience. Next, a small number of exercises will be conducted in pairs: drawing a lifeline, looking back at the past, sharing the strengths they have gained at their lowest moments, writing a “thank me letter,” and reading the letters to each other. They will discuss and share what they have learned and achieved through the program and the small, simple things they will continue to do for themselves and their families. The homework assignment is to use positive self-affirmation more often and give themselves a break to enjoy themselves.

#### Control group

Participants in the control group will attend discussion meetings led by a discussion leader, where they will be able to discuss their problems and share how they cope with them. No judgment or criticism will be given. After the two-month follow-up questionnaire, the control group will have the opportunity to participate in the same intervention. Partners will not participate in the discussion meetings.

### Babysitting support

A babysitting service, housed in a different room in the same building, will be available to participants of both groups upon request.

### Outcomes

#### Primary outcome

##### Resilience

Psychological resilience will be measured using the Psychological Resilience Scale [42], which consists of three subscales and a total of 21 items. Items are scored on a five-point Likert scale. Examples of items are “I’m curious about many things” (novelty pursuit); “I can control my anger” (ER); and “I believe I have a bright future” (positive future outlook). Higher scores indicate a higher degree of resilience.

#### Secondary outcomes

##### State-trait anger expression inventory

This widely used scale is a 44-item measure designed “to provide a means of measuring various components of anger” [43]. It includes the following subscales: state anger (how you feel now), trait anger (how you usually feel), anger-in (how you suppress anger), anger-out (how you express anger, aggression or insults to others), and anger control (how you control anger or do not express it or express it more appropriately). Since anger is felt differently when directed toward different people, we will assess anger toward both children and partners in terms of how it is expressed —i.e. anger-in, anger-out, and anger control.

##### Self-esteem

Self-esteem will be measured by the Japanese version of Rosenberg’s Self-esteem Scale [44]. This is a 10-item inventory with higher scores indicating a higher degree of self-esteem.

##### Parental attitude

The parenting attitude scale consists of five subscales measuring four negative and one positive attitude [45]. Examples of items are: “I can’t do what I want to do, I’m spending too much time on parenting” (burden of parenting), “I feel disgusted when children keep making a mess” (burden from children’s attitude and behavior), “I’m not confident about my parenting” (anxiety about parenting), “I’m afraid my child is more childish than other children” (anxiety about child’s development), and “I feel I’m growing through parenting.” (positive attitude toward parenting). Higher scores indicate a higher degree of each attitude.

##### Problem-focused coping strategies

The Problem-Focused Coping Strategies Scale [46] will be used to measure how caregivers cope with stressful situations. The measure consists of five subscales: solution calculation, concrete solution behavior, gathering information, goal orientation, and seeking solutions. We will use the first two scales, which will be enhanced for the purposes of this program.

*Family functioning* will be measured by the Japanese version of Family APGAR [47]. This measure consists of five parameters of family functioning: adaptability, partnership, growth, affection, and resolve. Items will be scored on a four-point scale, with higher scores indicating a higher degree of family functioning.

##### Cognition of children’s behavior

The Attribution on Children’s Misbehavior Scale consists of three subscales and measures the cognition of the attitudes and behavior of children [48]. Examples of items are: “I feel judged as ‘a bad parent’” (hostile attribution), “I struggle not knowing how to handle my child” (negative attribution), and “It’s a natural part of growing up” (positive attribution).

### Data collection

The outcome measures will be assessed online at the baseline, post-intervention, and at a two-month follow-up. Demographic data will be collected at the baseline and a program evaluation survey will be conducted post-intervention. Partners will be assessed (using the same measurement as for mothers) to evaluate the indirect impact from mothers sharing the knowledge and skills they gained in the sessions or discussions or seeing the changes in mothers’ attitude or behavior by participation.

### Statistical analysis

Data will be analyzed according to the intention-to-treat principle. The primary and secondary outcome measures will be analyzed at post-intervention and two-month follow-up using analysis of covariance to adjust for baseline differences between the groups. Subgroup analysis will be conducted using stratification factors including mother’s age, the area they live in, and number of children.

## Discussion

This paper describes the study protocol of a randomized controlled trial of a resilience-enhancement program for mothers, focused on emotion regulation. In contrast to previous parenting programs, which focus on teaching appropriate parenting skills, the novelty of this trial lies in its utilization of a CBT-based resilience-enhancement program for mothers for the purpose of the prevention of child-maltreatment. Our conceptual hypothesis is that the resilience enhancement training given to mothers in the intervention group will improve their psychological resilience and emotion regulation through the application of CR compared to mothers in the discussion group.

We further predict that, with these newly acquired skills, the intervention group will improve its level of self-esteem, family functioning and coping strategies and that this will have a positive impact on the families and children of these mothers.

If this program proves to be effective in enhancing resilience and ER among mothers, it may offer a new way to prevent maltreatment of children and protect the mental health of children and families.

## Data Availability

Not applicable.

## Abbreviations

CBT: cognitive behavioral therapy
CR: cognitive reappraisal
ER: emotion regulation
